# Effective Conservation Decisions Require Models Designed for Purpose: A Case Study of Boreal Caribou in Ontario's Ring of Fire

**DOI:** 10.1002/ece3.73199

**Published:** 2026-05-21

**Authors:** Matthew E. Dyson, Sarah Endicott, Craig Simpkins, Julie W. Turner, Stephanie Avery‐Gomm, Cheryl A. Johnson, Mathieu Leblond, Eric W. Neilson, Rob Rempel, Philip A. Wiebe, Jennifer L. Baltzer, Josie Hughes, Frances E. C. Stewart

**Affiliations:** ^1^ Biology Department Wilfrid Laurier University Waterloo Ontario Canada; ^2^ Wildlife and Landscape Science Directorate, Science and Technology Branch, Environment and Climate Change Canada Ottawa Ontario Canada; ^3^ University of British Columbia Vancouver British Columbia Canada; ^4^ Northern Forestry Centre, Canadian Forest Service Natural Resources Canada Edmonton Alberta Canada; ^5^ FERIT Environmental Consulting Thunder Bay Ontario Canada; ^6^ Great Lakes Forestry Centre, Canadian Forest Service Natural Resources Canada Sault Ste. Marie Ontario Canada

**Keywords:** caribouMetrics, decision‐support, demographic model, environmental impact assessment, open science, *Rangifer tarandus caribou*, resource selection function

## Abstract

Decision making in conservation science relies on the best available information. This may include using models that were not designed for purpose and are not accompanied by an assessment of limitations. To begin addressing these issues, we sought to reproduce and evaluate the spatial transferability of the two best available models for predicting impacts of proposed mining on boreal woodland caribou ( *Rangifer tarandus caribou* ) in northern Ontario. We evaluated their suitability for projecting the impacts of development in the Ring of Fire region. To aid in accessibility, we developed an R package for data preparation, modeling of resource selection, and demographic modeling. We found models were either ill‐suited or lacking for ongoing regional planning. The specificity of the regional resource selection model limited its usefulness for predicting impacts of development, and the high variability across caribou ranges limited the usefulness of a national aspatial demographic model for predicting range‐specific impacts. These existing models are not enough to provide spatially explicit information needed to minimize detrimental effects of anthropogenic development on caribou recovery in northern Ontario. Models designed for forecasting that are regularly updated with range‐specific demographic and habitat information are required.

## Graphical abstract

Decision making in conservation science often relies on the best available information, but existing models are rarely reproducible, transferrable, or accessible. We sought to reproduce and evaluate the suitability of the two best available models for predicting impacts of proposed mining on boreal woodland caribou ( *Rangifer tarandus caribou* ) in northern Ontario and evaluated their suitability for projecting the impacts of development in the Ring of Fire region. Neither model was sufficient to provide the spatially explicit information needed to minimize detrimental effects of anthropogenic development on caribou recovery in northern Ontario; models designed for forecasting are required.

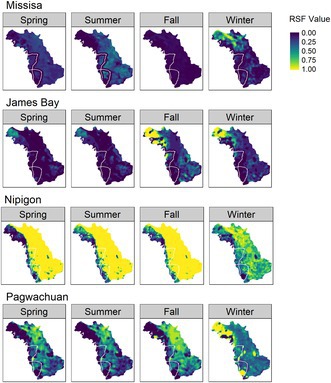

## 1 Introduction

Decisions about natural resource development should be informed by the best available information (Fuller et al. 2020). Models that quantitatively link wildlife species responses to development, such as wildlife‐habitat relationships and demography (Beyer et al. 2010; Matthiopoulos et al. 2020), can inform these decisions and associated policies (Wilson et al. 2021). However, these tools are generally not designed for decision support, and decision‐makers may be unaware of their limitations. For example, models are rarely reproducible; data and modeling processes are seldom reported nor accessible without author engagement, resulting in limited applicability outside of the specific context for which models were built. These problems can be addressed, in part, by implementing models in open source, and easily reproduced, decision‐support tools (e.g., Eacker et al. 2019; Nagy‐Reis et al. 2020; Nowak et al. 2018; McIntire et al. 2022; Dalgarno et al. 2025) that can inform impact assessment, particularly for Species at Risk (Roche et al. 2020).

**FIGURE 1:**
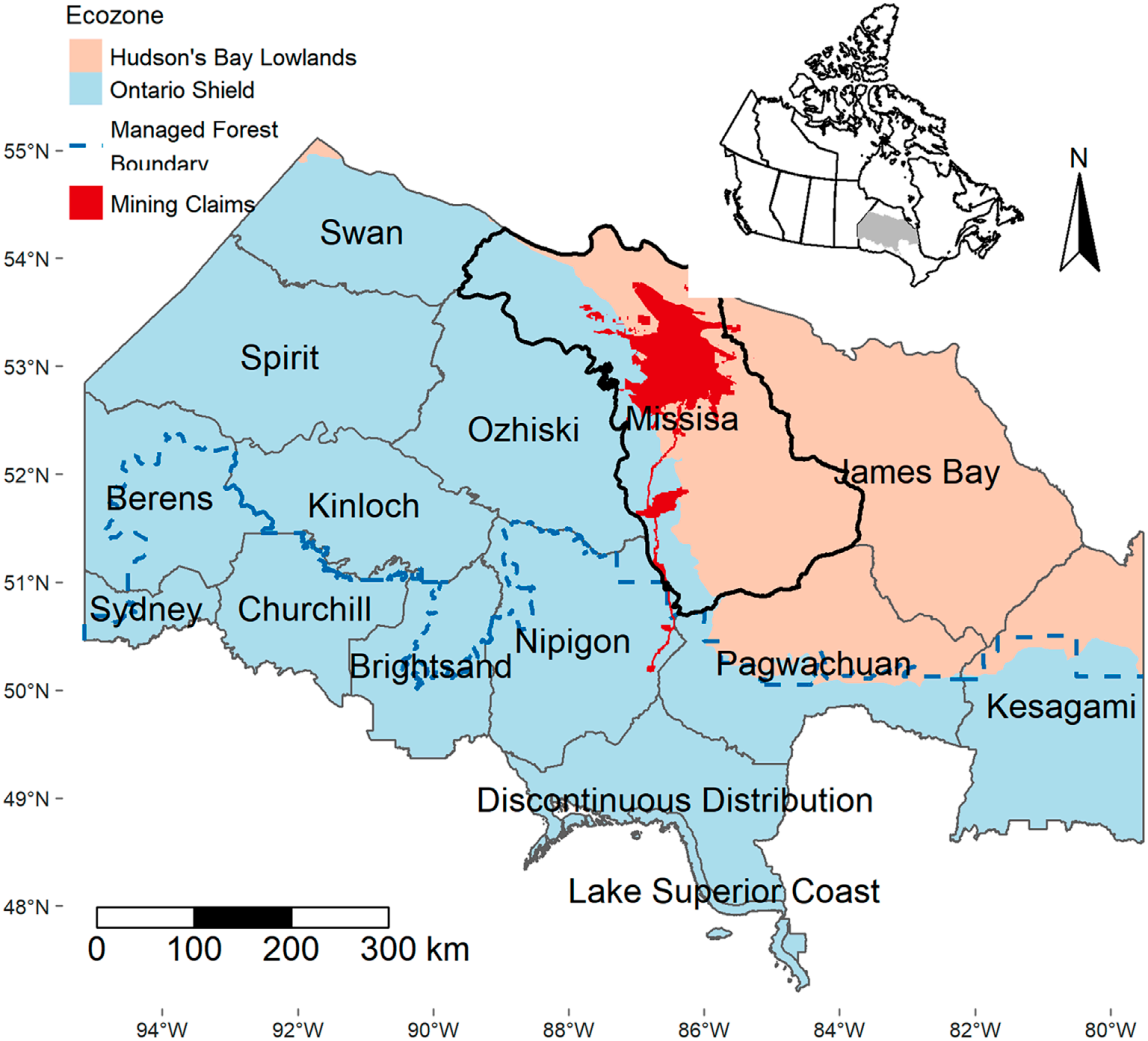
The Missisa boreal caribou range (black outline), which includes the proposed Ring of Fire (RoF) mining claims (dark red), is the focus of this study. The blue dashed line distinguishes the Managed Forest Zone (MFZ) from the Far North, and shading distinguishes the Ontario Shield ecozone (pale red) from the Hudson Bay Lowlands (blue). Inlay map (top‐right) indicates the location of the Ontario boreal caribou ranges (gray) relative to Canada.

As a country supporting some of the largest intact landscapes globally, Canada has a heightened responsibility to develop natural resources sustainably. For example, the Hudson Bay Lowlands of northern Ontario is one of the largest intact wetlands and peatland carbon stores on the planet (Ibisch et al. 2016; Poley et al. 2022; Sothe et al. 2022; Tootchi et al. 2019). The region is home to many wildlife species and is largely inaccessible by road. At the same time, it is rich in valuable minerals and is a target for resource extraction and economic development aimed at a green energy transition (Carlson and Chetkiewicz 2013; FNSAP 2011; IAAC 2021). With an ongoing Regional Assessment in the region (IAAC 2025), and much interest in critical minerals for batteries and other purposes (Natural Resources Canada), there is a need for modeling tools to assess the potential environmental impacts of the proposed mining of “Ring of Fire” (RoF) mineral deposits and associated development on wildlife (FNSAP 2011; IAAC 2021; Figure 1).

Quantitative knowledge of wildlife‐habitat relationships are commonly represented by resource selection functions (RSFs), which can produce maps of habitat suitability through space and time, that can be used to infer the consequences of habitat loss (Boyce et al. 2002; Harju et al. 2011; Johnson et al. 2004; Matthiopoulos et al. 2019). However, RSFs represent descriptive associations between the behavior of a sample of animals and habitat under scale‐specific conditions at the time the data were collected, potentially limiting their predictive capacity and transferability to novel conditions (Avgar et al. 2020; Kunegel‐Lion et al. 2022; Yates et al. 2018; Turner et al. 2025, but see Street et al. 2015). Aspatial demographic models are another tool that provide insight into drivers of population growth, and are also used for characterizing demographic responses to landscape alteration (Johnson et al. 2020; Sorensen et al. 2008; Stewart et al. 2020; Hughes et al. 2025a). However, high uncertainty about demographic parameters, drivers of change, and vital rates can limit the utility of such models for decision making and regional application (Chaudhary and Oli 2020; Sleep and Loehle 2010; but see Stewart et al. 2020 and Hughes et al. 2025a).

Boreal populations of woodland caribou ( *Rangifer tarandus caribou* ; hereafter “boreal caribou”), a well‐studied species, provide a useful test of the transferability of RSF and demographic models for understanding habitat loss. A species of conservation concern, boreal caribou inhabit large portions of Canada's boreal forest (ECCC 2011, 2019; Festa‐Bianchet et al. 2011), where anthropogenic disturbance threatens population persistence (Fryxell et al. 2020; Hebblewhite 2017; Stewart et al. 2020; Johnson et al. 2020). Nationally, only 29% of population ranges (15 of 51) are considered self‐sustaining (ECCC 2024a). In the province of Ontario, caribou are listed as threatened due to development from forestry and mineral exploration, including their road networks, which together increase predation and reduce adult female and calf survival (MNRF 2014a). Ongoing landscape disturbances are predicted to cause continued population declines (Fryxell et al. 2020). Effective conservation requires spatial and demographic tools that can project the effects of future landscape and climatic changes on caribou habitats and population performance. An existing Ontario RSF from adjacent caribou ranges (Hornseth and Rempel 2016) and a national demographic model derived from 58 areas spanning the species range (Johnson et al. 2020) represent the best available prediction tools in this region. Both have shortcomings (ECCC 2024b). Notably, they rely on a limited amount of data from over a decade ago, and their transferability to other contexts and their utility for projecting anticipated anthropogenic impacts have not been evaluated.

To advance conservation science decision making for a species at risk in a changing landscape, we sought to evaluate the suitability of the best available models for predicting the impacts of proposed mining in the RoF on Ontario's boreal caribou. The development of new models was not possible, until recently, due to data access restrictions. Therefore, we reproduced existing caribou RSF and demographic models and evaluated their suitability for spatial transferability and prediction of potential impacts of proposed mining in the RoF. Models based on species at risk data can be difficult to fully reproduce as information is rarely made publicly available. We demonstrate and discuss the challenges of this exercise and address some of those challenges with open‐source tools and reproduceable workflows in an R package (Wickham 2015; caribouMetrics) that can be integrated into predictive frameworks to support resource‐use decisions (Bodner, Rauen Firkowski, et al. 2021; McIntire et al. 2022).

## 2 Methods

### 2.1 Study Area

Boreal caribou range in Ontario is delineated into 13 local populations (Fig. 1; ECCC 2024). We focused on four caribou ranges that are adjacent to or overlap with the RoF mining claims and had published RSF coefficients: Missisa, James Bay, Pagwachuan, and Nipigon. The Missisa range contains most of the RoF mineral claims (FNSAP 2011; IAAC 2021) and is the focus of our study. Missisa and James Bay ranges occur in Ontario's Far North (FNSAP 2011), where anthropogenic disturbance has so far been limited; Nipigon and Pagwachuan ranges are almost entirely within the Managed Forest Zone (MFZ) where industrial forestry is common (Figure 1).

These four ranges straddle the Ontario Shield and Hudson Bay Lowlands ecozones (Figure 1). The Shield consists of mixed and coniferous forests dominated by black spruce ( *Picea mariana* ), and containing jack pine ( *Pinus banksiana* ), balsam fir ( *Abies balsamea* ), white spruce ( *Picea glauca* ), trembling aspen ( *Populus tremuloides* ), and balsam poplar ( *Populus balsamifera* ; Crins et al. 2009). Missisa and Pagwachuan ranges include the transition between the Shield and Lowlands, which is composed of peatland complexes with poor drainage, including black spruce and tamarack ( *Larix laricina* ; Crins et al. 2009). The James Bay range is almost entirely within the Lowlands and the Nipigon range is entirely within the Shield (Figure 1).

### 2.2 RSF Model Reproduction and Validation

We reproduced existing RSFs for these four caribou ranges using tables of published boreal caribou RSF coefficients from these same areas as Hornseth and Rempel (2016; hereafter referred to as the “original RSFs”; and Rempel et al. 2021). The data used to produce the original RSFs were collected between 2009 and 2013 from GPS‐collared caribou across a variety of companion studies within the ranges of interest; however, these data are not publicly available and error estimates for model coefficients were not published (Hornseth and Rempel 2016; MNRF 2014a, 2014b; Rempel and Hornseth 2018). We acquired data sets for the predictor variables that were publicly available and matched the data sources described by Hornseth and Rempel (2016) as closely as possible. Details on model reproduction, predictor data, an assessment of model reproducibility, and validation can be found in Supporting Information (Part 1).

### 2.3 Disturbance Scenarios and RSF Model Projection

To assess the suitability of existing RSFs for projecting the impacts of disturbance on boreal caribou habitat selection we considered three simple disturbance scenarios: (i) a “base” scenario with no change in disturbance footprint (0.41% anthropogenic disturbance), (ii) a “roads‐only” scenario that included proposed RoF access roads (1.11% anthropogenic disturbance; MNDMNRF 2022), and (iii) a “roads‐and‐mines” scenario that included the proposed access roads and mining claims associated with the RoF (16.91% anthropogenic disturbance; Table 1; Figure S1.1). The original RSF included road density as a predictor and required additional information on roads within mining areas that we were not able to obtain; we therefore only compared the base and road‐only scenarios for the RSF but included all three scenarios for the demographic model (see below).

**TABLE 1:** Summary of changes in buffered anthropogenic disturbance and fire, excluding anthropogenic disturbance (as defined in Johnson et al. 2020), in three scenarios: The base scenario without additional development, roads only scenario, and roads‐and‐mines scenario that included the proposed roads and mining claims within the Ring of Fire area in the Missisa caribou range.

Scenario	Anthropogenic (%)	Fire (%)	Total disturbance (%)	Fire excluding anthropogenic (%)
Base	0.41	4.27	4.58	4.17
Roads only	1.11	4.27	5.23	4.12
Roads‐and‐mines	16.91	4.27	20.60	13.69

To understand how the “roads‐only” scenario might affect resource selection, we projected the original RSFs across updated landscape conditions as represented by (i) temporal changes in forest structure between 2010 and 2020 (e.g., including simulated fires) and (ii) the new proposed roads‐only scenario (Table 1; Figure S1.1). We also assessed the potential for borrowing RSF information from other ranges by transferring coefficients from the top original RSFs from adjacent ranges (James Bay, Nipigon, and Pagwachuan) to Missisa. We visualized the spatial transferability of these models and examined their sensitivity to changing habitat availability using scatterplots where we compared the value of the model response for each grid cell produced by the Missisa model and each respective adjacent range.

### 2.4 Demographic Model Reproduction, Validation, and Projection

We reproduced Canada's national demographic boreal caribou model (Johnson et al. 2020) to predict changes in survival (*S*) and recruitment (*R*) within the roads‐only and roads‐and‐mines disturbance scenarios. Code and regression model parameters were made available upon request from the national demorgraphic models (Johnson et al. 2020). We calculated the relevant predictor variables for the Missisa range based on Johnson et al. (Johnson et al. 2020; i.e., % anthropogenic disturbance buffered by 500 m; % wildfire within the last 40 years; Table 1), and calculated expected survival and reproduction ($\bar{S}$, $\bar{R}$) as a function of disturbance according to the beta regression models with highest support (M4 and M1 respectively from Johnson et al. 2020). We characterized the distribution of outcomes for our three disturbance scenarios (Table 1; Figure S1.1) and report both the expected population growth rate $\bar{\lambda }=\bar{S}\left(1+\bar{R}/2\right)$ (Hatter 2020; Hatter and Bergerud 1991; Hughes et al. 2025a) and realized growth rate that includes interannual variation, demographic stochasticity, and density dependence (λ). To verify our reproduction of the demographic model used by Johnson et al. (2020), we compared our outputs to those from model code supplied by the authors. Details on model reproduction, evaluation, incorporating among‐population variability in demographic rates, projection of within‐range demographic uncertainty, and model validation can be found in Supporting Information (Part 2).

### 2.5 caribouMetrics: An R Package for Boreal Caribou Modeling

We incorporated RSF and demographic model components into the *caribouMetrics* R package with documentation and vignettes explaining their use (Hughes et al. 2025b). We used GitHub to promote version control and transparency of the development process.

## 3 Results

### 3.1 RSF Model Reproduction, Validation, and Projection

We were able to produce a reasonably comparable representation of the original RSFs for the Missisa range (Hornseth and Rempel 2016), as evidenced by high Pearson's *r* values across all seasons (*r* > 0.935), but some locations showed different predictions depending on the season (Figures S1.2 and S1.3). This was expected given the input data was not exactly the same between the two methods (Figure S1.4). In the Missisa range, the model predicted the highest relative probabilities of use in the northwest of the study area during winter (Figure S1.6), consistent with the original RSFs. During the spring and summer, the eastern portion of the range had a higher relative probability of use compared to the northwest (Figure S1.5).

**FIGURE 2:**
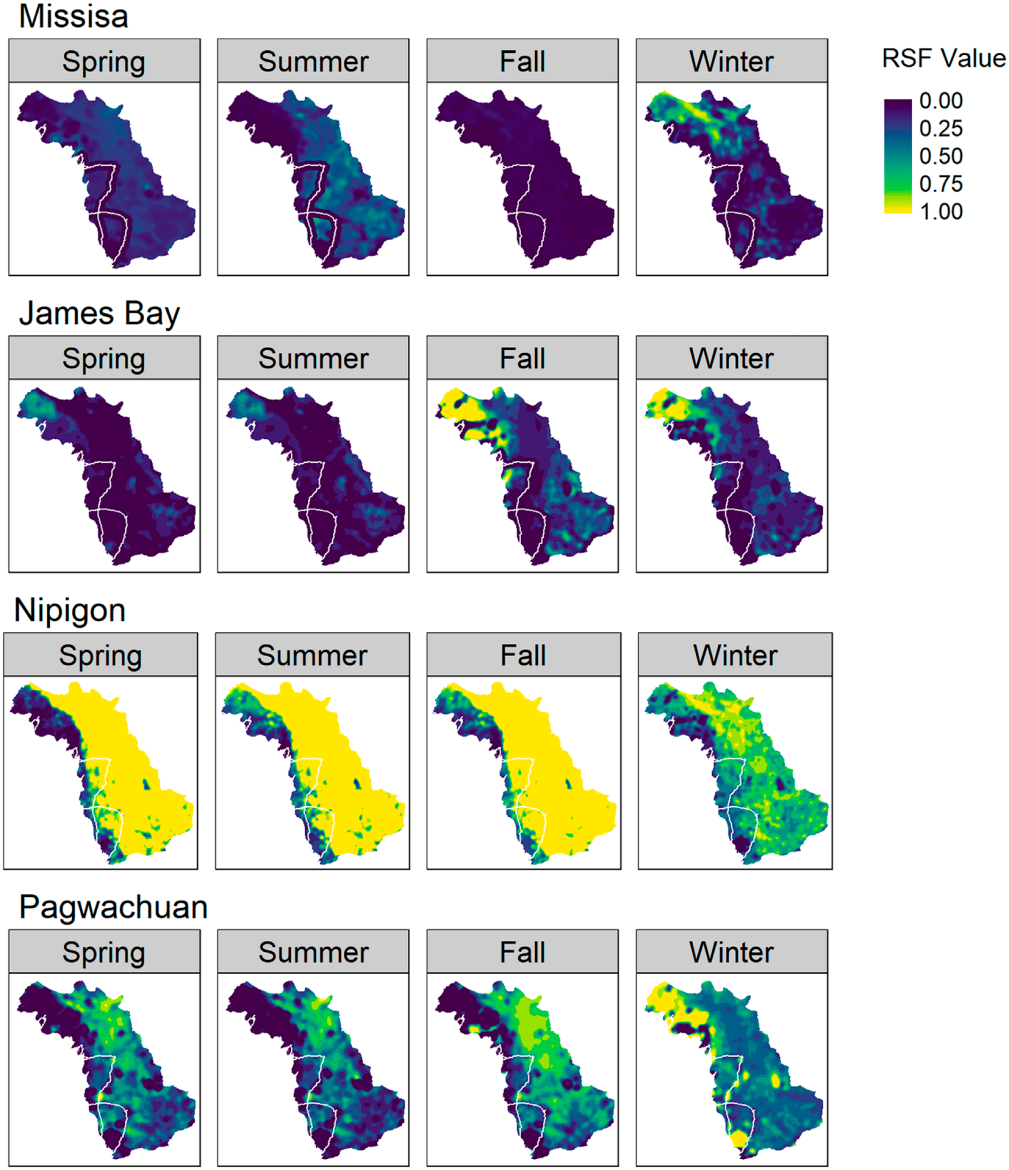
Seasonal RSF predictions from *caribouMetrics* in the Missisa range under the roads only scenario using the coefficients from Hornseth and Rempel (2016) from the Missisa, James Bay, Nipigon, and Pagwachuan range to estimate the relative probability of use (0–1) by boreal caribou during spring, summer, fall, and winter. Scale ranges from dark blue to yellow with yellow representing a higher relative probability of use. Proposed roads are represented by white lines.

We observed a consistent lower relative probability of use in areas associated with proposed roads in the spring and summer (Figure 2, Figure S1.6), consistent with seasonal changes in the response to roads described in the original RSFs. There was high variability in the estimated response to roads among ranges; the James Bay prediction appeared the most similar to the Missisa prediction; however, the discrepancy varied by season. Nipigon and Pagwachuan projections visually differed from the Missisa projection and did not show a marked negative response to proposed roads (Figure 2).

### 3.2 Demographic Model Reproduction, Validation, and Projection

**FIGURE 3:**
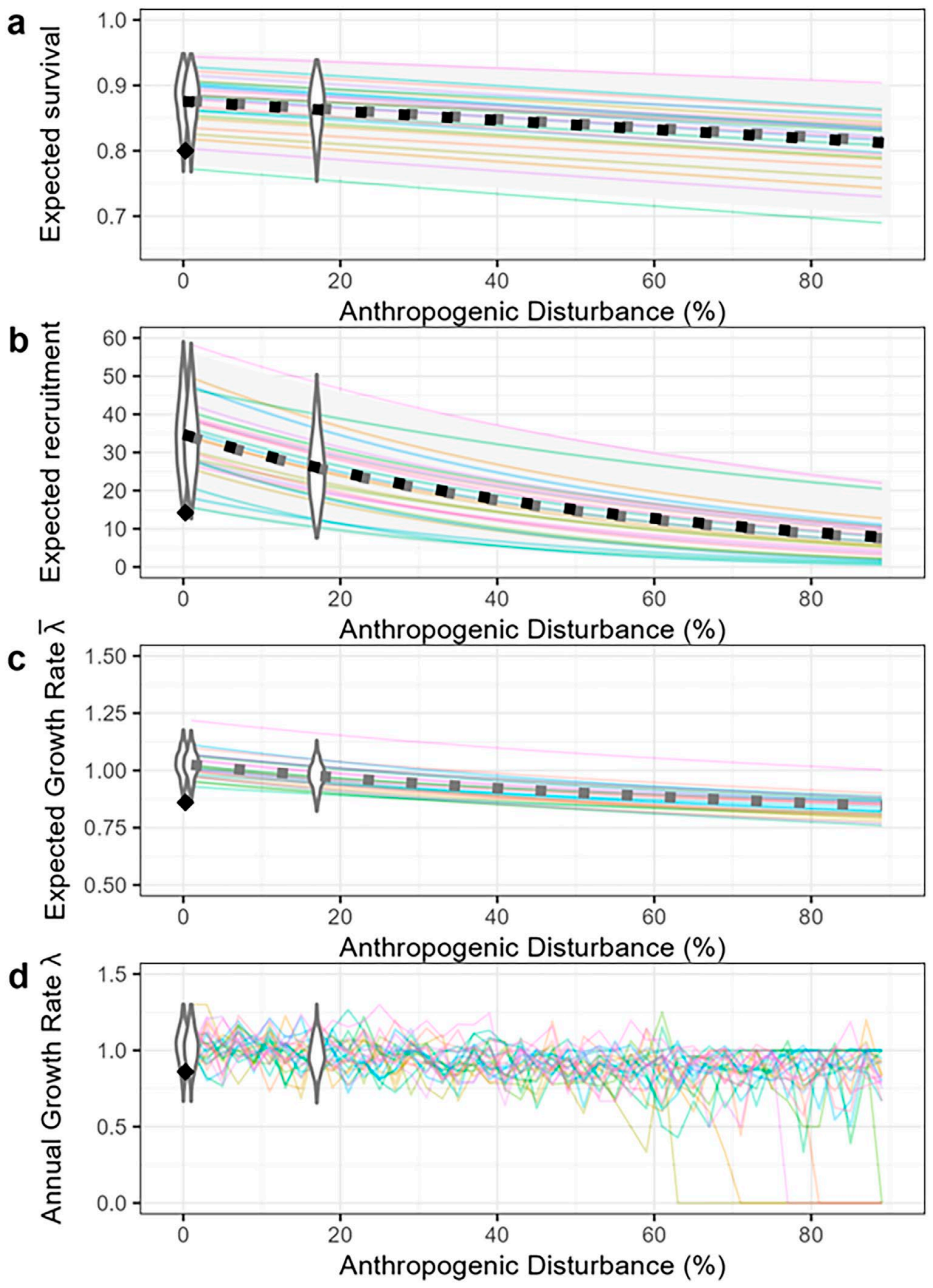
Demographic rate simulations derived from regression models in Johnson et al. (2020) for (a) expected adult female survival (S), (b) expected recruitment (R; calves per 100 cows), (c) expected population growthrate (λ), and (d) population growth rate with interannual variation (λ). Overlap of gray and black dotted lines in (a) and (b) indicates a good match between expected values from our model (gray boxes) and Johnson et al. (2020) (black boxes). Colored lines show effects of changing anthropogenic disturbance on demographic rates in 25 sample populations, assuming sample populations are randomly distributed among quantiles of the beta distribution, and each population remains in the same quantile of the beta distribution as disturbance changes. Alignment of these sample trajectories with 95% predictive intervals from Johnson et al. (2020) (pale gray bands in panels a and b) indicates that we have adequately reproduced variability in that model. Violin plots show the distribution of outcomes for 500 sample populations under the three disturbance scenarios for the Missisa range (from left to right, Base, Road Only, and Roads and Mines). The black diamond indicates the demographic rates for the Missisa range according to a 2014 assessment (MNRF 2014a). Populations where λ < 0.99 are considered not self‐sustaining.

Our regression models for survival and recruitment closely matched those of Johnson et al. (2020). Anthropogenic disturbance remained low in all our disturbance scenarios (Table 1), and the corresponding range of variability in demographic rates among sample populations was high (Figure 3). The model predicted increasing anthropogenic disturbance would decrease both survival and recruitment, but the importance of that decrease for self‐sustainability of the population was highly uncertain and depended on initial population status (Figure 3).

## 4 Discussion

We examined the applicability of two kinds of models and assessed their ability to describe effects of anticipated land use change on boreal caribou behavior and demography in the context of impact assessment. We found that the original RSF for the Missisa boreal caribou range was poorly suited for projecting impacts of disturbance; likely because it was too specific to regional conditions at the time of original model development when disturbance was limited (Hornseth and Rempel 2016; Avgar et al. 2020; Rempel et al. 2021). This led to considerable uncertainty about the impact of linear features (e.g., roads), as their effect varied greatly among regions and seasons with most ranges showing an order of magnitude lower effect size than Missisa. These factors, combined with the region‐specific modeling approach, may have caused the RSFs to underestimate future impacts of linear features. In addition, an existing boreal caribou demographic model was too general to project the impacts of disturbance on this same boreal caribou range. Additional data collection in the region is ongoing (ECCC 2024a, 2024b) but local data alone will not be sufficient to project impacts of disturbance in a region that remains largely undisturbed. There is a need for new models that are better suited to forecasting the impacts of disturbance in northern Ontario.

### 4.1 Lessons Learned From Using Non‐Predictive Models to Forecast Impacts

Across caribou ranges where anthropogenic disturbance is low, the national demographic model predicted high variation in recruitment and survival among populations (Figures 3 and 5 of Johnson et al. 2020), leading to imprecision and uncertainty in projected impact of increasing disturbance on population growth rate in the RoF (Figure 3 here). This variability highlights the need for local information about the current (i.e., baseline) demographic parameters of caribou populations in the RoF and ongoing monitoring of disturbance impacts in the area (Eacker et al. 2019). An assessment of the status of the Missisa population based on 2008–2012 data (MNRF 2014b) suggested survival, reproduction, and population growth rate in this range were lower than predicted by the national demographic model of Johnson et al. (2020) (see black diamond in Figure 3). The national demographic model (Johnson et al. 2020) predicts that increasing anthropogenic disturbance will decrease both survival and recruitment, but the importance of that decrease for population viability depends on baseline demographic parameters. Even small changes in adult female survival can affect the sustainability of a population when survival is low (Johnson et al. 2020).

### 4.2 Modeling Challenges and Opportunities

The original RSF models (Hornseth and Rempel 2016) were fit independently with data from each range. This is a reasonable approach when the objective is to characterize current habitat use, but not for projecting responses to changing landscape conditions (Wan et al. 2019; Winter et al. 2024). In the original RSFs there was high variability in the effect of linear features among range‐specific RSFs, suggesting that the behavioral response of caribou to linear features may vary with the amount of disturbance in a range (i.e., a functional response; Mysterud and Ims 1998), or that effects of linear features were confounded with other correlated predictors (e.g., forest harvest). In addition, the response to linear features likely varies within or between feature types (e.g., road or pipeline) requiring careful consideration during model parameterization (Wolfe et al. 2000, Dyson et al. 2022). Hierarchical regional models that include functional responses to disturbance (Matthiopoulos et al. 2011; Avgar et al. 2020; Muff et al. 2020; Olson et al. 2021; Teitelbaum et al. 2021) could yield models that are better suited for projection. Our method of modeling variation in survival and recruitment among populations (Figure 3) adequately reproduced the observed variation among populations (Johnson et al. 2020). We assumed demographic parameters vary with disturbance according to the best supported national models, but note that other competing models with comparable support are also plausible and might yield somewhat different projections when applied to northern ranges with low anthropogenic disturbance (*sensu* Stewart et al. 2023). The simple population model we used is highly relevant to conservation practice for boreal caribou in Canada (ECCC 2011, 2019, 2024b), but a more thorough investigation of the sensitivity of caribou demographic projections to variation in model form and measurement errors is warranted. We anticipate that methods for integrating national demographic‐disturbance relationships with local survival and recruitment information will help reduce uncertainty in range‐specific demographic projections (see Hughes et al. 2025a).

### 4.3 Next Steps for Supporting Actionable Science in the Ring of Fire Region

The RoF region itself provides a modeling challenge as it is extremely remote. Existing data and our associated understanding of the region is lacking. Even for well‐studied species, such as caribou, there are substantial knowledge gaps compounded by uncertainties in the disturbance history, impacts, and recovery of peatlands and other land cover types that will affect them. There are various regional efforts underway to collect data needed to inform resource development and wildlife management decisions (e.g., Government of Canada 2022), and we are hopeful about opportunities for collaborative development of region‐specific open‐source forecasting models. More data, including GIS data, may be needed in the RoF region, but it does not follow that more data is needed in all regions and circumstances. We advocate for an open, transparent, iterative modeling approach that allows for integration of data and models as they become available, *sensu* caribouMetrics (see Eacker et al. 2019 and Hughes et al. 2025a) . These models should be designed for purpose, account for and map uncertainty, be updateable with new information (e.g., local data), and be transparent, simple, and reproducible (Bodner, Brimacombe, et al. 2021). This approach allows ongoing assessment of the implications of additional data for model uncertainty while addressing important questions about what additional information is required to inform conservation decisions.

## 5 Conclusions

We reiterate a call for improvements in future models, and their workflows, used for conservation decision‐making (Eacker et al. 2019; Bodner, Brimacombe, et al. 2021). We encourage developers of wildlife response models and collectors of relevant wildlife data to work together toward this goal (Davidson et al. 2021; Russell et al. 2021). Approaches include (i) working in multi‐disciplinary teams (Bodner, Brimacombe, et al. 2021), (ii) recognizing that steps and training toward transparency and reproducibility are valuable, even if the result is not always an easily usable tool, and (iii) acknowledging code does not have to be flawless to enable others to build on previous work. In this project, we were able to reproduce existing models only because the developers of those models were willing to share covariate data and code, and to discuss model generation procedures. A shift to open workflows will reduce the need to ask for this information, reduce the chance of errors, increase efficiency, and advance our ability to make informed decisions for conservation science and practice.

## Author Contributions

**Matthew E. Dyson:** conceptualization (equal), data curation (equal), formal analysis (equal), investigation (equal), methodology (equal), visualization (supporting), writing – original draft (lead), writing – review and editing (equal). **Sarah Endicott:** data curation (equal), formal analysis (equal), methodology (equal), visualization (equal), writing – review and editing (equal). **Craig Simpkins:** data curation (equal), formal analysis (supporting), methodology (supporting), writing – review and editing (supporting). **Julie W. Turner:** methodology (supporting), writing – review and editing (equal). **Stephanie Avery‐Gomm:** writing – review and editing (supporting). **Cheryl A. Johnson:** formal analysis (supporting), methodology (supporting), supervision (supporting), writing – review and editing (supporting). **Mathieu Leblond:** supervision (supporting), writing – review and editing (supporting). **Eric W. Neilson:** supervision (supporting), writing – review and editing (supporting). **Rob Rempel:** formal analysis (supporting), methodology (supporting), software (supporting), writing – review and editing (supporting). **Philip A. Wiebe:** conceptualization (supporting), methodology (supporting), writing – review and editing (supporting). **Jennifer L. Baltzer:** conceptualization (equal), funding acquisition (equal), project administration (equal), supervision (equal), writing – review and editing (supporting). **Josie Hughes:** conceptualization (equal), data curation (supporting), formal analysis (equal), funding acquisition (equal), investigation (equal), methodology (equal), project administration (equal), software (equal), supervision (equal), visualization (supporting), writing – original draft (supporting), writing – review and editing (supporting). **Frances E. C. Stewart:** conceptualization (equal), formal analysis (supporting), funding acquisition (equal), investigation (supporting), methodology (supporting), project administration (equal), supervision (equal), writing – original draft (supporting), writing – review and editing (supporting).

## Funding

This work was supported by the Canadian Wildlife Service and the Wildlife and Landscape Science Directorate of Environment and Climate Change Canada. F. E. C. Stewart and J. L. Baltzer were supported by the NSERC Canada Research Chairs Program.

## Conflicts of Interest

R.R. is the principal of FERIT Consulting. F.E.C.S. and J.L.B. were project leads at Wilfrid Laurier University, and J.H. was a project lead at ECCC.

## Supporting information


**Data S1:** ece373199‐sup‐0001‐Supinfo.docx.

## Data Availability

The version of the caribouMetrics R package used in this paper is available at https://github.com/LandSciTech/caribouMetrics/tree/EcoEvoMissisaPaper, and analysis code is available at https://github.com/LandSciTech/MissisaBooPaper. Data required for analysis is available in this OSF repository: https://osf.io/r9mkp/?view_only=fb71321265d14dbeb3d932e4de66be0c.
