# Supplementary material for: Effective Conservation Decisions Require Models Designed for Purpose: A Case Study of Boreal Caribou in Ontario's Ring of Fire
Source: Ecol Evol. 2026 May 21;16(5):e73199. doi: 10.1002/ece3.73199 (PMC13240247; doi:10.1002/ece3.73199)
Supplement: Supplementary file 1 — Data S1: ece373199‐sup‐0001‐Supinfo.docx. [file ECE3-16-e73199-s001.docx]

Supplementary Material for

**Effective conservation decisions require models designed for purpose: a case study for boreal caribou in Ontario’s Ring of Fire**

[*Insert reference here, removed to blind the review process*]

**Part 1.** **Boreal Caribou Resource Selection Model Reproduction and Validation**

To reproduce existing RSFs for caribou in northern Ontario we used tables of published boreal caribou RSF coefficients for the same areas as Hornseth and Rempel (2016) (hereafter referred to as the ‘original RSFs’). Hornseth and Rempel referred to these models as resource selection probability functions (RSPFs), but their approach differs from what is commonly understood as an RSPF (Johnson et al., 2006); as such we use the term ‘RSF’ throughout. The data used to produce the original RSFs were collected between 2009 and 2013 from GPS-collared caribou across a variety of companion studies within the ranges of interest; however, these data are not publicly available and error estimates for model coefficients were not published (Hornseth and Rempel, 2016; MNRF, 2014a, 2014b; Rempel and Hornseth, 2018). Instead, we obtained the published coefficients for range-specific seasonal RSFs of each reported top model (Table S1.1) for the Nipigon, Pagwachuan, Missisa, and James Bay ranges. However, associated error or uncertainty information for individual coefficients was not reported in Hornseth and Rempel (2016) and model performance was only reported at the model level (e.g., sensitivity, specificity, and area under the receiver operating characteristic [ROC] curve).

To assess our ability to reproduce the original models, we acquired the original projected surfaces for comparison from the authors (R. Rempel pers. comm, 2021). We validated our models visually and quantitatively. The original models used a nested hexagon grid, common in Ontario (e.g., Poley et al. 2014; Ray et al. 2018), to generate predictions. For ease of future implementation using raster tools, we approximated this approach with distance-weighted moving windows on a rectangular grid, and compared the two approaches by extracting the values from our rectangular grid to the 592-hectare hexagonal grid provided by the authors using the weighted mean. We mapped the difference in predictions and explanatory variables between the models (Fig S1.2) and used scatterplots to compare the values of the model responses for each grid cell produced by both methods (Fig. S1.3). A perfect reproduction would produce a Pearson correlation coefficient of 1, and any deviation from the original prediction would reduce this value. We expected some differences in the predictions to result from the type of grid and predictor data sets used.

We validated our reproduction of the (Hornseth and Rempel, 2016) RSF model by comparing our results to the results of the original model which were provided by the authors (R. Rempel pers. Comm, 2021) and using recently published coefficients (Table S1.1; Rempel et al., 2021). To reproduce the results we acquired data sets for the predictor variables that were as close as possible to those used by Hornseth and Rempel (Table S1.2, S1.3). Despite our best efforts some data sets were no longer available so we used available data to approximate them. The original provincial land cover and eskers data sets were available and we assumed that no new railways were built in the study area. The disturbance dataset used by Hornseth and Rempel was not available so we used fire data available from the Ontario Ministry of Natural Resources and Forestry (OMNRF) and filtered it to include disturbances up to 2010 (Table S1.2). There is no commercial forestry in the Missisa range so we did not include harvest data. Similarly, only current road and utility data was available so these were used and then filtered to linear features built before 2010 or for which no date was provided. These predictor variables were used as inputs to the caribouHabitat function in the caribouMetrics R package to calculate the relative probability of use by boreal caribou in each season (Hughes et al. 2025b; Fig S1.5).

**Table S1.1.** Coefficients used in RSF models to analyze habitat use by caribou in northern Ontario (Rempel et al., 2021). Confidence intervals were not provided by the original authors so uncertainty of the coefficients is not included.

| **Range** | **Variable** | **Spring** | **Summer** | **Fall** | **Winter** |
| --- | --- | --- | --- | --- | --- |
| Nipigon | Dense Deciduous | -44.8817 | -24.0817 | -38.5131 | -2.10533 |
|  | Sparse Conifer | -1.61879 | 2.76126 | 1.10202 | 3.26042 |
|  | Linear Features | -0.0591871 | -0.108422 | -0.0657281 | -0.107917 |
|  | Gravel Esker | 0.445199 | 0.724132 | 0.351276 | -0.428307 |
|  | Dense Conifer | 0.63021 | -0.902747 | 0.61502 | 1.16347 |
|  | Natural Burn | 0.775091 | 1.1134 | 1.44648 | -2.84384 |
|  | Open Water | 2.33635 | -0.120702 | 1.2464 | -0.969895 |
|  | Mixed | 7.39955 | 2.87828 | 4.35131 | 2.07816 |
|  | Conifer Peatland | 7.82448 | 4.37304 | 7.98351 | 0.960811 |
|  | Open Peatland | 20.2548 | 26.2326 | 27.1499 | 6.35273 |
|  | Intercept | -3.19835 | -2.46477 | -2.81546 | -1.81318 |
| Pagwachuan | Natural Burn | -42.0006 | -41.3792 | -34.2672 | 23.8352 |
|  | Dense Deciduous | -37.6389 | -104.387 | -39.0287 | -3.48497 |
|  | Mixed | -4.58361 | -1.83914 | -14.1546 | 3.51487 |
|  | Sparse Conifer | -4.5621 | -2.01557 | -13.7814 | 15.4934 |
|  | Conifer Peatland | -2.7476 | 0.360641 | -6.26648 | 10.9739 |
|  | Linear Features | -0.35184 | -0.123711 | -0.738744 | -0.59024 |
|  | Open Water | 1.40204 | 7.37853 | -6.11699 | 16.6945 |
|  | Dense Conifer | 1.6818 | 5.30014 | -6.47953 | 9.68799 |
|  | Gravel Esker | 3.55455 | 2.24184 | 2.86622 | 5.2854 |
|  | Open Peatland | 4.39236 | 7.53761 | -3.48516 | 10.5729 |
|  | Intercept | -0.15753 | -3.73633 | 6.50218 | -11.7408 |
| Missisa | Linear Features | -47.0453 | -54.014 | -1.1584 | -32.4414 |
|  | Natural Burn | -7.00107 | -1.09398 | -3.85024 | 4.07806 |
|  | Sparse Conifer | -3.20956 | 6.03112 | 4.23322 | 10.5872 |
|  | Conifer Peatland | -2.00532 | 12.3221 | 1.31976 | -2.54278 |
|  | Dense Conifer | -1.26999 | 12.1113 | 3.02311 | 1.38101 |
|  | Open Peatland | -0.252612 | 7.91777 | 4.61501 | 10.8757 |
|  | Open Water | 0.690524 | 9.73417 | 4.23221 | -1.55981 |
|  | Mixed & Deciduous | 1.65949 | -9.0874 | -11.7367 | -4.96709 |
|  | Intercept | -0.223601 | -10.9329 | -5.98728 | -5.28692 |
| James Bay | Mixed & Deciduous | -8.12364 | -14.0371 | -15.4255 | -0.851921 |
|  | Open Peatland | -6.76679 | -6.2329 | -9.0392 | 3.65392 |
|  | Dense Conifer | -5.14577 | -4.58141 | -15.0418 | -4.66829 |
|  | Open Water | -0.871053 | -1.01115 | -11.1888 | -3.62043 |
|  | Linear Features | -0.0389879 | -0.0964143 | -9.69171 | 0.0889229 |
|  | Natural Burn | 0.783623 | -0.917044 | -18.8983 | -4.8709 |
|  | Sparse Conifer | 2.6777 | 1.2406 | 3.07579 | 11.3358 |
|  | Conifer Peatland | 3.41592 | 2.80308 | -6.80688 | 0.559626 |
|  | Intercept | -1.79609 | -1.09174 | 6.65858 | -3.18369 |

**Table S1.2.** Descriptions and sources of the spatial data used to parameterize models of caribou resource selection in Ontario. The versions of these datasets used in this analysis are available at <https://osf.io/r9mkp/>

| **Data set** | **Variable** | **Description** | **Source** |
| --- | --- | --- | --- |
| Fire disturbance area | DTN | Fires > 40 ha. Filtered to include fires from the preceding 30 years (i.e., 1980-2010 for comparison to the original model and 1990-2020 for the ring of fire projections) | <https://geohub.lio.gov.on.ca/datasets/fire-disturbance-area> |
| MNRF road segments and Ontario road network | TDENLF | Roads in both datasets that did not fully overlap were combined by removing roads from the Ontario road network that were within 100 m of a road in the MNRF data. Roads present in 2010 were approximated based on roads that had a year of construction in the MNRF data before 2010 or had no year of construction | <https://geohub.lio.gov.on.ca/datasets/mnrf-road-segments> and <https://geohub.lio.gov.on.ca/datasets/mnrf::ontario-road-network-orn-segment-with-address> |
| Ontario railway network | TDENLF | All railways were assumed to be have been present in 2010 | <https://geohub.lio.gov.on.ca/datasets/mnrf::ontario-railway-network-orwn> |
| Utility line | TDENLF | Utility lines present in 2010 were approximated by filtering the data by the “business effective date” | <https://geohub.lio.gov.on.ca/datasets/m> nrf::utility-line |
| Quaternary Geology | ESK | Extracted esker line features | <https://www.mndm.gov.on.ca/en/mines-and-minerals/applications/ogsearth/quaternary-geology> |
| Provincial Land Cover (2000) | DEC, CON, MIX, LGW, ST, LGTP, LGOP, LGMD | Landsat derived land cover data with 27 classes | <https://geohub.lio.gov.on.ca/datasets/provincial-land-cover> |
|  |  |  |  |
| ROF mine area | Anthropogenic Disturbance | Used to reflect anthropogenic disturbance in the Roads and Mines scenario. For simplicity, only mining claims associated within the ring of fire (crescent) and associated large area to the south found within the database were used in scenarios. Identifying the proportion of claims that are mined and the extent of mining within a claim was outside the scope of this work. | <https://www.ontario.ca/page/mining-lands-administration-system> |
| ROF proposed roads | TDENLF | Combined with existing roads for the Roads Only scenario. The proposed roads were a polygon feature derived from the mining data converted to a line feature to calculate density. | <https://www.ontario.ca/page/mining-lands-administration-system> |
| Caribou range boundary |  | Used to clip data to Missisa range boundary | <https://geohub.lio.gov.on.ca/datasets/caribou-range-boundary> |
| Ecozones |  | Used for mapping study area | <https://sis.agr.gc.ca/cansis/nsdb/ecostrat/gis_data.html> |
| Missisa model output from Hornseth and Rempel (2016) |  | Output by the original model authors used to validate our reproduction of the model | Rob Rempel pers comm 2021 |
| MNR Forest Management Units (FMUs) |  | Used for mapping study area. FMUs were unioned to get the outline of the AOU | <https://geohub.lio.gov.on.ca/datasets/lio::forest-management-unit/about> |

**Table S1.3.** Predictor variables included in RSF models to analyze habitat use by caribou in northern Ontario. Described in Hornseth and Rempel (2016).

| **Variable** | **Code** | **Description** | **Measurement** |
| --- | --- | --- | --- |
| Dense deciduous | DEC | Provincial land cover class | Proportion |
| Mixed deciduous and conifer | MIX | Provincial land cover class | Proportion |
| Open peatland | LGOP | Combination of open fen and open bog provincial land cover classes | Proportion |
| Dense conifer | CON | Provincial land cover class | Proportion |
| Conifer peatland | LGTP | Provincial land cover class | Proportion |
| Mixed + deciduous | LGMD | Sum of MIX and DEC | Proportion |
| Sparse conifer | ST | Sparse forest provincial land cover class | Proportion |
| Open water | LGW | Combination of open water and turbid water provincial land cover classes | Proportion |
| Natural burn | DTN | Natural disturbance based on fire | Proportion |
| Gravel esker | ESK | Natural linear feature resulting from glacial deposits | Density (m/ha) |
| Linear Features | TDENLF | Human-built linear features, including roads, railways, and hydro transmission lines | Density (m/ha) |

**
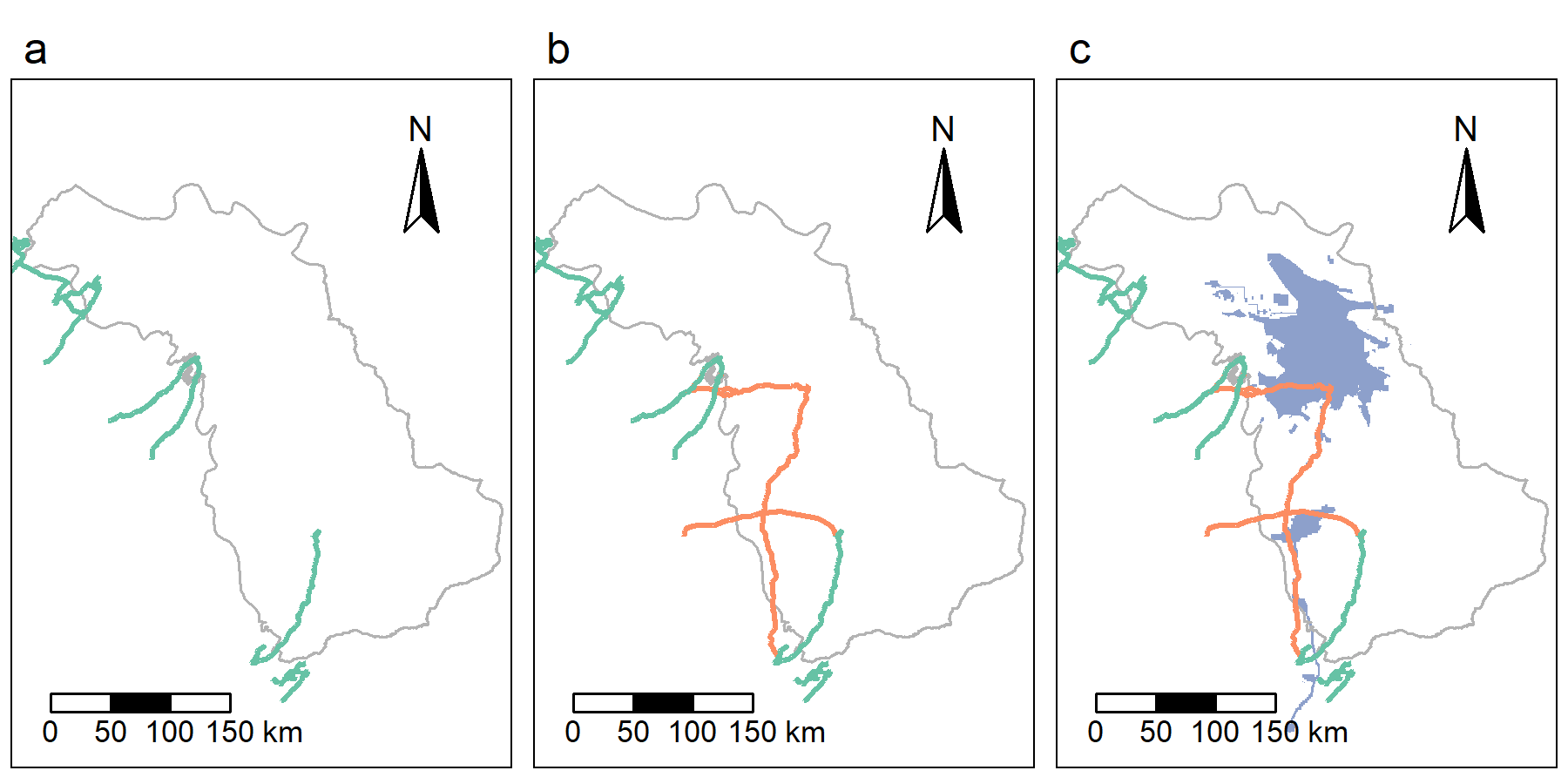
**

**Figure S1.1.** Maps of the Missisa range with the extent of linear features included in (a) the original model developed by Hornseth and Rempel (base), (b) the roads-only scenario used to project the RSF model, and (c) the roads-and-mines scenario used for demographic modelling. Existing roads are represented as green lines, proposed roads as orange lines, and mines are coloured in purple.

*
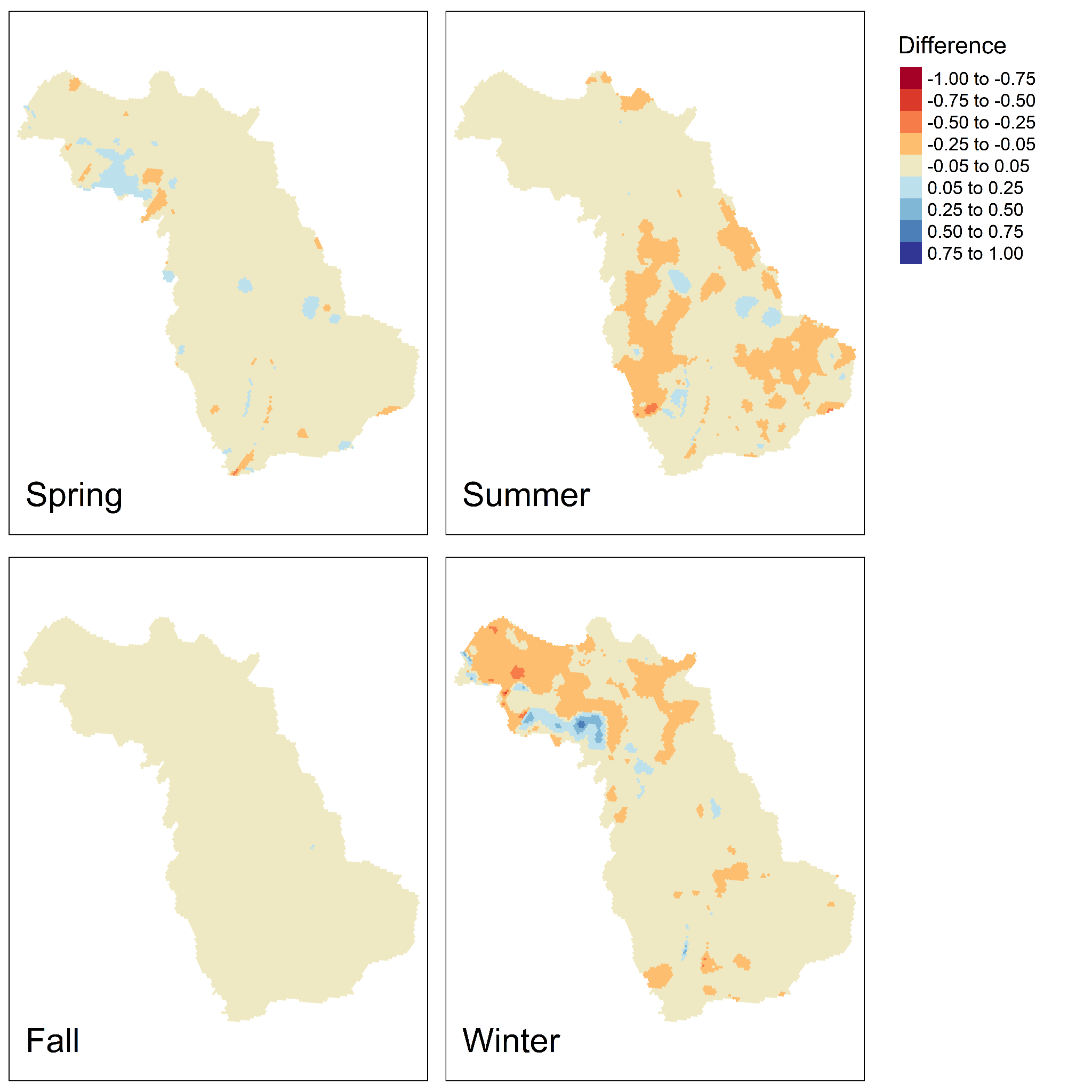
*

**Figure S1.2.** Difference in modelled resource selection probability results from *caribouMetrics* in the Missisa range between Hornseth and Rempel (2016) and our reproduction of their models across all seasons. Negative values (warm tones) are areas where our mean selection coefficients were higher than Hornseth and Rempel (2016) and positive values (cool notes) are areas where they are lower.

*
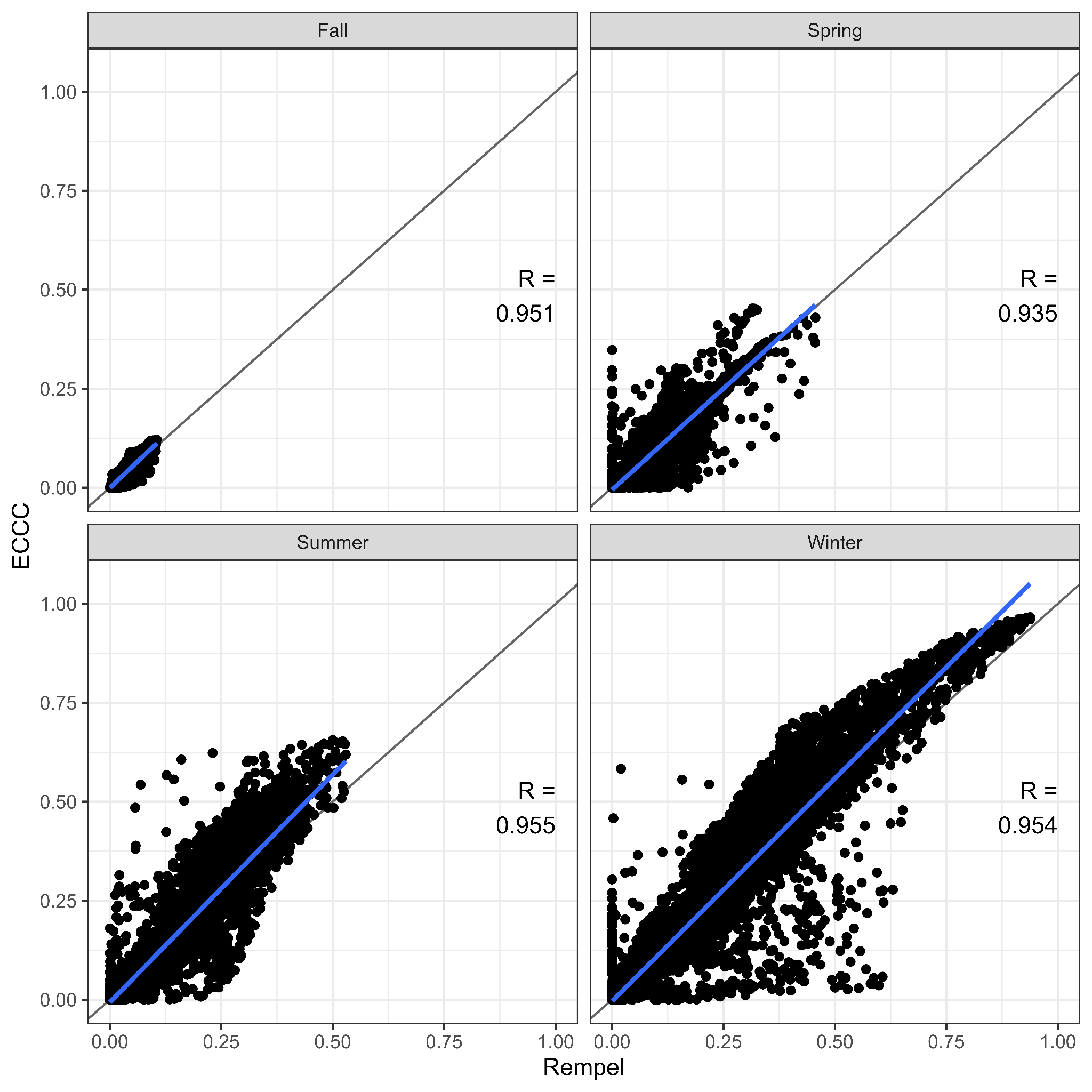
*

**Figure S1.3.** Comparison of pixel values between the original model published by Hornseth and Rempel (2016; Rempel, x-axis) and our reproduction (ECCC, y-axis) for the four seasons (Spring, Summer, Fall, Winter). Pearson’s R values are provided in each plot as a measure of correlation as well as the estimated regression line (blue).

*
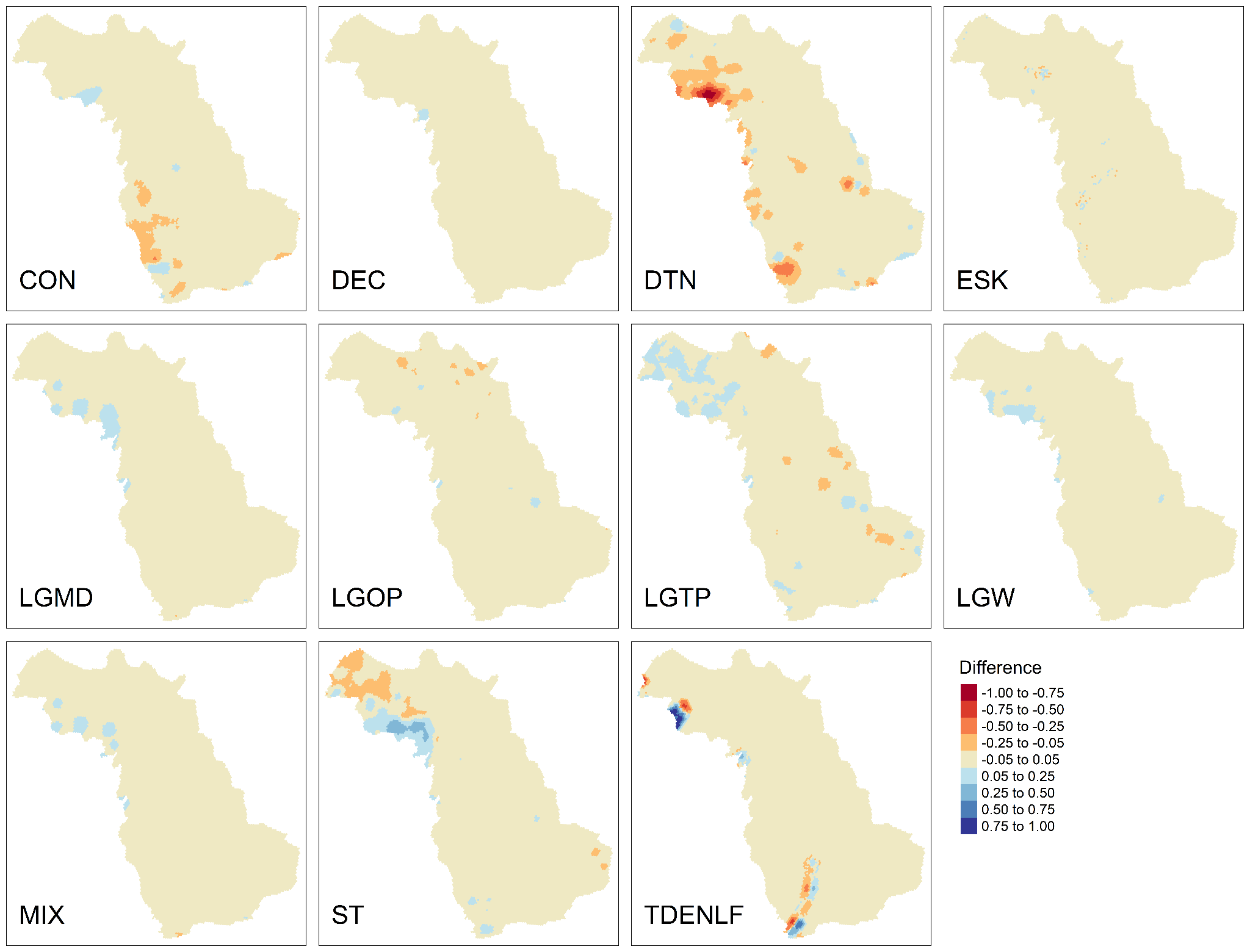
*

**Figure S1.4.** Differences in predictor variables used by Hornseth and Rempel (2016) and in this analysis. Negative values (warm tones) are areas where our predictor values are higher than Hornseth and Rempel’s, and positive values (cool tones) are areas where they are lower. Predictor variable names are defined in Table S1.3. Natural burn, linear features, and sparse conifer had the greatest difference when comparing the covariate plots between the original model and the data used for our exercise (here and in Figure S1.5).

**Figure S1.5**. Reproduction of the seasonal RSF for the Missisa range from Hornseth and Rempel (2016) using caribouMetrics and the published coefficients to reproduce the relative probability of use (0-1) by boreal caribou during spring, summer, fall, and winter. The predictor variables used are approximations of those used by Hornseth and Rempel (2016) based on currently available data. Scale ranges from dark blue to yellow with yellow representing a higher relative probability of use.
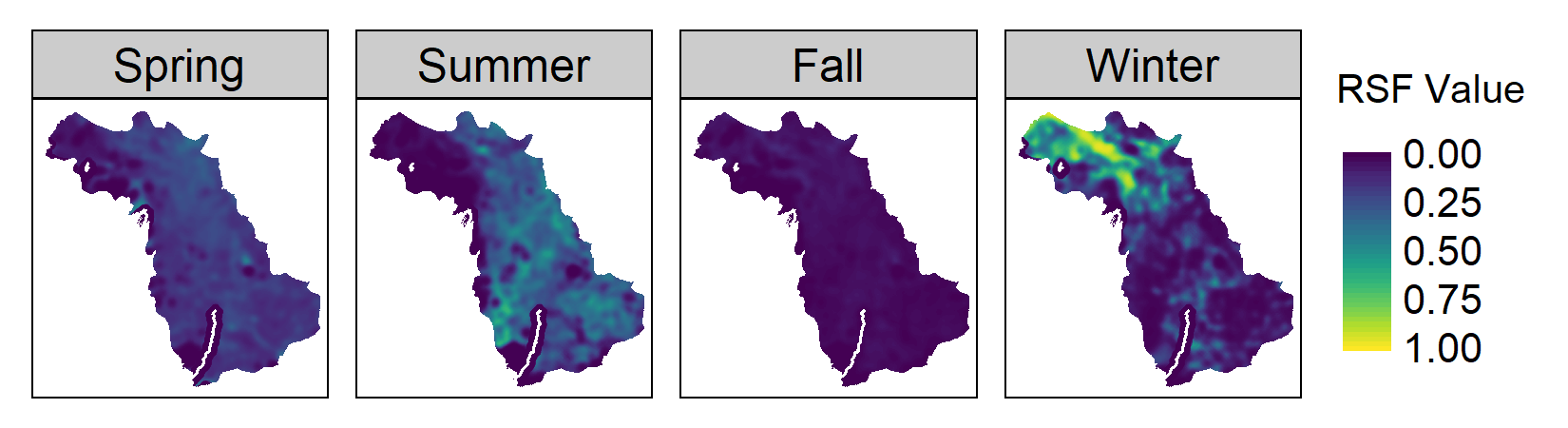


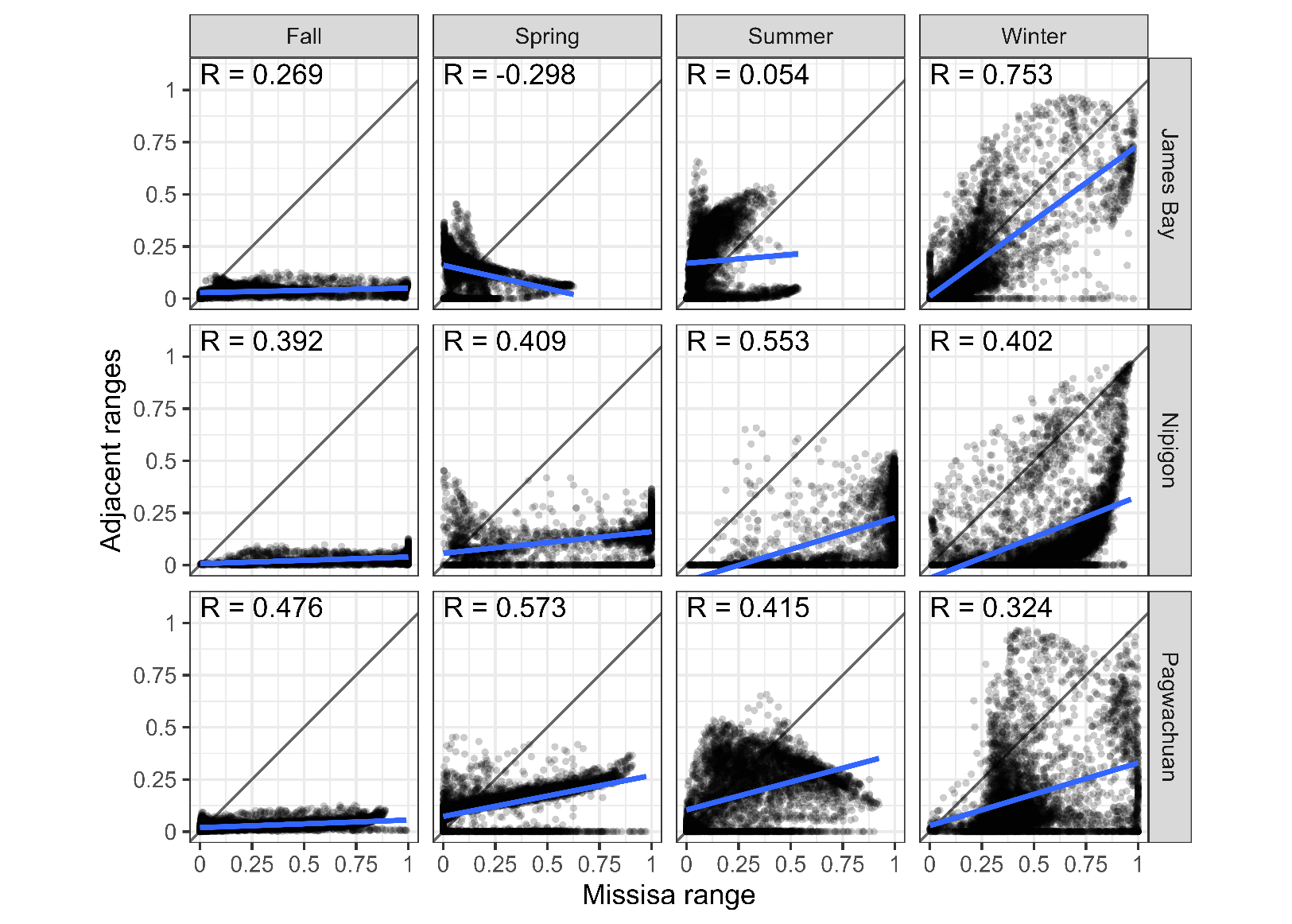


**Figure S1.6.** Comparison of the seasonal RSF predictions from *caribouMetrics* in the Missisa range under the roads only scenario using the coefficients from Hornseth and Rempel (2016) for the Missisa range and using coefficients for each of the James Bay, Nipigon, and Pagwachuan ranges to estimate the relative probability of selection (0-1) by boreal caribou during spring, summer, fall, and winter. Pearson’s R values are provided in each plot as a measure of correlation, where 1 would represent a perfect correlation. The blue line shows the fit of a linear model and the diagonal line represents a perfect reproduction.

**References**

Hornseth, M.L., Rempel, R.S., 2016. Seasonal resource selection of woodland caribou (*Rangifer tarandus caribou* ) across a gradient of anthropogenic disturbance. Can. J. Zool. 94, 79–93. <https://doi.org/10.1139/cjz-2015-0101>

Johnson, C.J., Nielsen, S.E., Merrill, E.H., Trent, L., Boyce, M.S., Science, E., Program, M., British, N., George, P., 2006. Resource Selection Functions Based on Use – Availability Data : Theoretical Motivation and Evaluation Methods. Journal of Wildlife Management 70, 347–357. <https://doi.org/10.2193/0022-541X(2006)70>

MNRF, 2014a. State of the Woodland Caribou Resource Report. Ministry of Natural Resources and Forestry - Species at Risk Branch, Thunder Bay, ON.

MNRF, 2014b. Integrated Range Assessment for Woodland Caribou and their Habitat - The Far North of Ontario 2013 (No. Version 1.1). Ministry of Natural Resources and Forestry - Species at Risk Branch, Thunder Bay, ON.

Poley, L.G., Pond, B.A., Schaefer, J.A., Brown, G.S., Ray, J.C. and Johnson, D.S., 2014. Occupancy patterns of large mammals in the Far North of Ontario under imperfect detection and spatial autocorrelation. *Journal of Biogeography*, *41*(1), pp.122-132.

Ray, J.C., Poley, L.G., Magoun, A.J., Chetkiewicz, C.L.B., Meg Southee, F., Neil Dawson, F. and Chenier, C., 2018. Modelling broad‐scale wolverine occupancy in a remote boreal region using multi‐year aerial survey data. *Journal of biogeography*, *45*(7), pp.1478-1489.

Rempel, R.S., Hornseth, M.L., 2018. Range-specific seasonal resource selection probability functions for 13 caribou ranges in Northern Ontario (No. IFR-01), Science and Research Internal File Report. Ministry of Natural Resources and Forestry, Peterborough, ON.

**Part 2: Demographic Model Description, Validation, and Reproduction**

Canada’s national demographic boreal caribou model was developed from adult female survival, calf recruitment, and landscape data across 58 boreal caribou study areas, including 13 study areas in Ontario (Johnson et al., 2020). It is a two-stage model, which we used to predict changes in survival and recruitment within the roads-only and roads-and-mines disturbance scenarios (Table 1, Fig. S1.3). The national demographic model is aspatial and all types of anthropogenic disturbances are combined into a single measure of disturbed area within a range; using the ‘roads-and-mines’ scenario is sufficient and there is no need to specify the location of roads within mining claims. We calculated the relevant predictor variables for the Missisa range based on Johnson et al. (2020; i.e., % anthropogenic disturbance buffered by 500 m; % wildfire within the last 40 years; Table 1), and calculated expected recruitment ($R_{t}$) and survival ($S_{t}$) as a function of disturbance according to the beta regression models with highest support (M4 and M1 respectively from Johnson et al., 2020):

$$R_{t}\sim Beta\left( \mu_{t}^{R},\phi^{R} \right);log\left( \mu_{t}^{R} \right)=\dot{\beta}_{0}^{R}+\dot{\beta}_{a}^{R}A_{t}+\dot{\beta}_{f}^{R}F_{t}, \left( eq 1a \right)$$

$$S_{t}\sim\left( 46\times Beta\left( \mu_{t}^{S},\phi^{S} \right)-0.5 \right)/45;log\left( \mu_{t}^{S} \right)=\dot{\beta}_{0}^{S}+\dot{\beta}_{a}^{S}A_{t}. \left( eq 1b \right)$$

$\phi^{R}\sim\text{Normal}\left( 19.862,2.229 \right)$ and $\phi^{S}\sim\text{Normal}\left( 63.733,8.311 \right)$ are precisions of the Beta distributed errors (Ferrari and Cribari-Neto, 2004), and survival rates are back transformed as in Johnson et al. 2020. Table 3 of Johnson et al. (2020) provides the expected values and 95% confidence intervals of all regression coefficients ($\dot{\beta}_{0}^{R}, \dot{\beta}_{a}^{R},\dot{\beta}_{f}^{R},\dot{\beta}_{0}^{S}, \dot{\beta}_{a}^{S})$, which are assumed to be Gaussian distributed. To evaluate these recruitment (eq 1a) and survival (eq 1b) models, we sampled expected demographic rates across a range of anthropogenic and fire disturbance (0-100%) to reproduce expected values and 95% predictive intervals from Fig. 3 and Fig. 5 of Johnson et al. (2020).

In areas of low anthropogenic disturbance, such as the Missisa and James Bay ranges, there is substantial among-population variability in recruitment (Fig. 3 in Johnson et al., 2020). To model this variation, we selected regression model parameter values for each study area (i.e., a sample population) at the beginning of simulations and assigned each to quantiles of the error distributions for survival and recruitment. Sample populations remained in their quantiles as the landscape changed, allowing us to distinguish the effects of changing disturbance from variation in initial population status. To show these effects, we projected population growth for 35 sample populations across a wide range of anthropogenic disturbance levels (0-90%). We slightly altered Johnson et al’s (2020) demographic model by including demographic stochasticity (see Hughes et al. 2025a for details). Specifically, we noted an issue with rounding of small numbers, leading to an overestimate of lambda when initial population sizes and demographic rates are both low (Fig. S2.1 and Fig. S2.2). Here, we report results from a modified version of the demographic model that uses binomial sampling rather than rounding in order to allow small populations to go extinct:

$$W_{t}\sim binomial(N_{t},S_{t}),$$

$m_{t}={[p}_{0}-(p_{0}-p_{K})\left( \frac{W_{t}}{N_{0}K} \right)^{b}]\frac{W_{t}}{W_{t}+a}$,

$J_{t}\sim binomial(W_{t},sR_{t}m_{t}$),

$$N_{t+1}={min(W}_{t}+J_{t},r_{max}N_{t}),$$

where $N_{t}$is the number of adult females at time *t*, $W_{t}$is the number of adult females that survive to breeding, $S_{t}$ is adult female survival, $J_{t}$ is the number of recruits to the adult female class, $R_{t}$is recruitment rate measured as calf:cow ratio, and $m_{t}$determines how recruitment varies with population density (Lacy et al., 2017). Given the parameter values in Table S2.1, recruitment rate is lowest ($0.25R_{t}$) when $N_{t}=1$, approaches a maximum of $0.5R_{t}$ for intermediate population sizes, and declines to $0.3R_{t}$as the population reaches carrying capacity of 100 times the initial population size $N_{0}.$

Following Johnson et al., (2020) we model interannual variability in recruitment and survival using truncated beta distributions (rtrunc function; Novomestky and Nadarajah, 2016), with mean value for each sample population from the regression models (eq 1a, eq 1b). We characterized the distribution of outcomes for our three disturbance scenarios (Table 1; Fig S1.3) more thoroughly by projecting demographic rates for 500 sample populations. Population growth rate each year is given by *λ_t_=N_t+1_/N_t_* when *N_t_>0* and *λ_t_=0* when *N_t_=0*. Expected population growth rate without interannual variation, demographic stochasticity, or density dependence is $\lambda_{t}=S_{t}\left( 1+R_{t}/2 \right)$ (Hatter 2020; Hatter and Bergerud 1991; Hughes et al. 2025a). To show the effects of changing anthropogenic disturbance we projected the growth of 500 sample populations over a 45 year period as anthropogenic disturbance increased from 1 to 90%. We assumed an initial population size of 373 females, which we derived from the minimum animal count of 745 caribou between 2009 and 2011, assuming 50% were female (MNRF, 2014b). This estimate is conservative because adult male:female ratios are typically lower in caribou (ECCC, 2011; Espinoza and Weckerly, 2021). Coefficients of variation among years and maximum/minimum values for recruitment and survival are given in Table S2.1. We report both the expected growth rate $\lambda_{t}$ and the realized growth rate *λ_t_*. To verify our reproduction of the demographic model used by Johnson et al. (2020), we compared our outputs to those from model code supplied by the authors.

The demographic modelling workflow is implemented in the *caribouMetrics* R package, and described in the Caribou Demography vignette: https://landscitech.github.io/caribouMetrics/articles/caribouDemography.html [(Hughes et al. 2025b)](https://landscitech.github.io/caribouMetrics/reference/popGrowthJohnson.html).

The standardized nature of packages allows them to easily integrate into the larger R ecosystem, facilitating ease of extension and adaptation for tasks beyond their original design (e.g. prediction or forecasting; McIntire et al., 2022). caribouMetrics also contains functions that automate the geospatial data preparation process to facilitate application to new landscapes. The original RSFs were developed using a closed scripting language called Landscape Scripting Language (LSL; Kushneriuk and Rempel, 2011), which we re-implemented in R. For the demographic model, we began by borrowing demographic rate sampling code from a SpaDES (Chubaty and McIntire, 2022) module (caribouPopGrowth; <https://github.com/tati-micheletti/caribouPopGrowthModel>; Stewart et al., 2023), and advanced the method to include precision and quantiles. Together, this makes the national caribou demographic model (Johnson et al. 2020) more transparent, reproducible, and adaptable to local population estimates.

**Table S2.1.** Demographic model parameter values.

| Model Parameter | Description | Value |
| --- | --- | --- |
| $p_{0}$ | Maximum recruitment multiplier. | 1 |
| $p_{K}$ | Recruitment multiplier at carrying capacity. | 0.6 |
| $K$ | Carrying capacity multiplier. | 100 |
| $b$ | Density dependence shape parameter. | 4 |
| $a$ | Allee effect parameter. | 1 |
| $s$ | Sex ratio. | 0.5 |
| $r_{max}$ | Maximum population growth rate. | 1.3 |
| $v_{R}$ | Coefficient of variation in $R_{t}$among years. | 0.2116 |
| $v_{S}$ | Coefficient of variation in $S_{t}$among years. | 0.0076 |
| $l_{R}$ | Minimum value for $R_{t}.$ | 0 |
| $h_{R}$ | Maximum value for $R_{t}.$ | 0.82 |
| $l_{S}$ | Minimum value for $S_{t}.$ | 0.61 |
| $h_{S}$ | Maximum value for $S_{t}.$ | 1 |


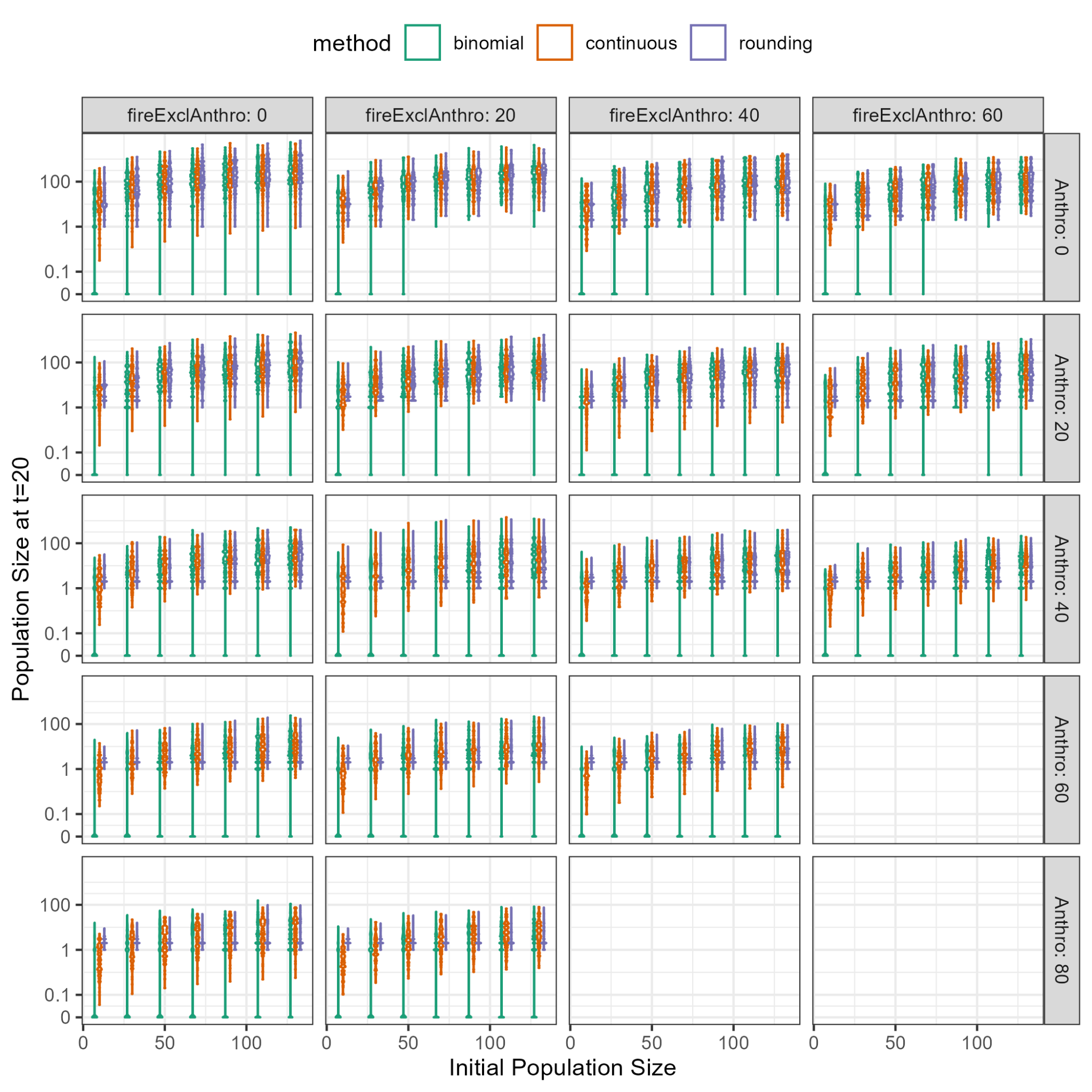


**Figure S2.1.** Effects of the method for obtaining whole numbers of animals on population size. Violin plots show the distribution of outcomes among 100 survival and recruitment samples for each combination of fire, anthropogenic disturbance, and initial population size. The “rounding” method used by Johnson et al., (2020) does not allow populations to go extinct, leading to an overestimate of persistence probability when initial population sizes and demographic rates are both low. The “continuous” method allows partial animals, and the “binomial” method samples the number of survivors and recruits from a binomial distribution.

**
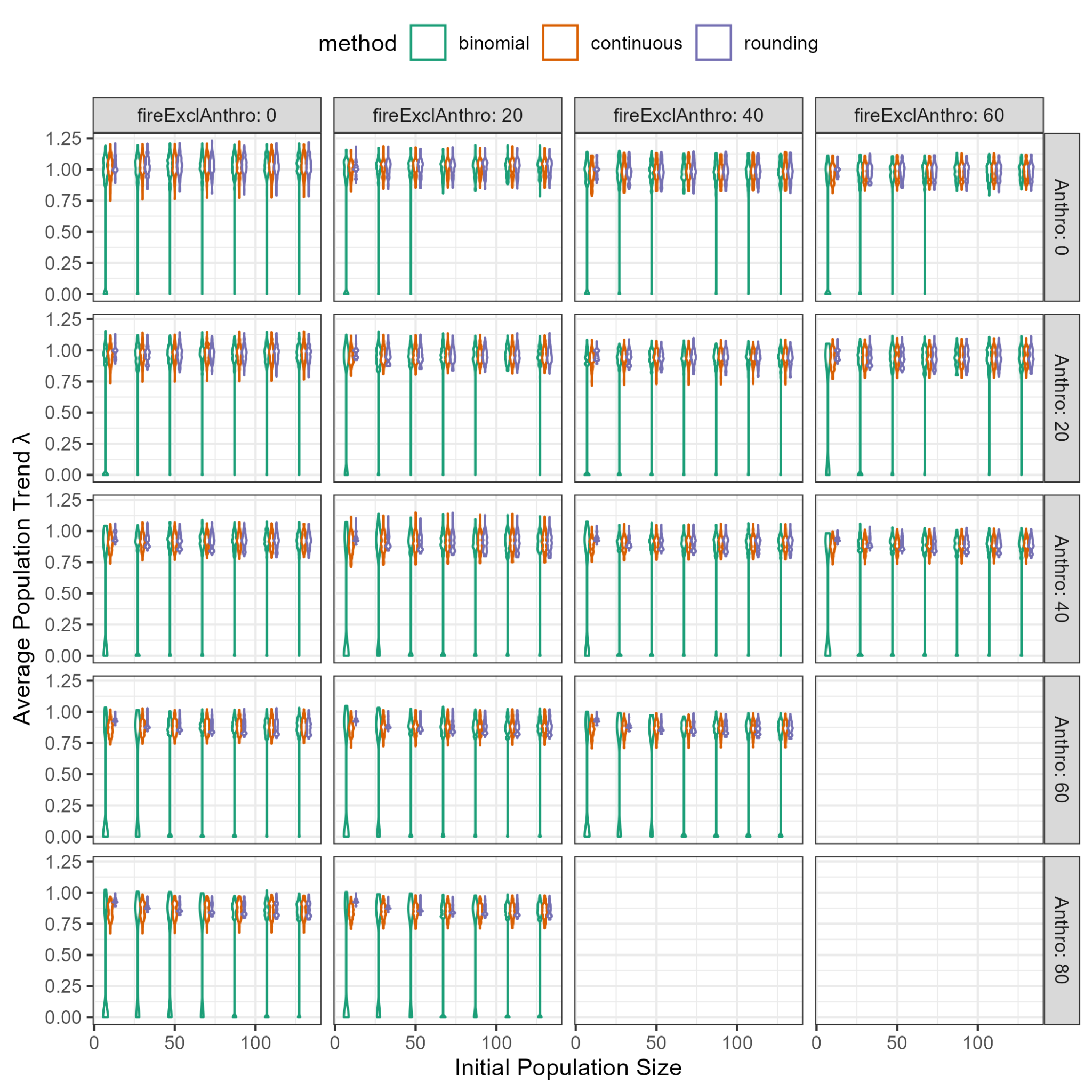
**

**Figure S2.2.** Effects of the method for obtaining whole numbers of animals on realized population growth rate. Violin plots show the distribution of the geometric mean of realized population growth rate over 20 years among 100 survival and recruitment samples for each combination of fire, anthropogenic disturbance, and initial population size. The “rounding” method used by Johnson et al., (2020) does not allow populations to go extinct, leading to an overestimate of population growth when initial population sizes and demographic rates are both low. The “continuous” method allows partial animals, and the “binomial” method samples the number of survivors and recruits from a binomial distribution.

**References**

Chubaty, A.M., McIntire, E.J.B., 2022. SpaDES: Develop and Run Spatially Explicit Discrete Event Simulation Models.

ECCC, 2011. Scientific assessment to inform the identification of critical habitat for woodland

caribou (Rangifer tarandus caribou), boreal population, in Canada. Canadian Wildlife

Service, Ottawa.

Espinoza, Z.S., Weckerly, F.W., 2021. A Comparative Analysis of Adult Sex Ratios in Polygynous and Monogamous Mammal Populations. amid 186, 299–308. https://doi.org/10.1674/0003-0031-186.2.299

Ferrari, S., Cribari-Neto, F., 2004. Beta Regression for Modelling Rates and Proportions. Journal of Applied Statistics 31, 799–815. https://doi.org/10.1080/0266476042000214501

Hornseth, M.L., Rempel, R.S., 2016. Seasonal resource selection of woodland caribou ( *Rangifer tarandus caribou* ) across a gradient of anthropogenic disturbance. Can. J. Zool. 94, 79–93. <https://doi.org/10.1139/cjz-2015-0101>

Hughes, J., Endicott, S., Calvert, A.M., and Johnson, C.A. 2025. Integration of national demographic-disturbance relationships into local caribou population viability projections can reduce uncertainty and inform monitoring decisions.*Ecological Informatics* 87*.* <https://doi.org/10.1016/j.ecoinf.2025.103095>

Hughes J., Endicott S., Shimoda Y., Simpkins C., Michelleti T., McIntire E. 2025b. caribouMetrics: Models and Metrics of Boreal Caribou Demography and Habitat Selection. R package version 0.4.0.9001, <https://github.com/LandSciTech/caribouMetrics/tree/EcoEvoMissisaPaper>

Johnson, C.A., Sutherland, G.D., Neave, E., Leblond, M., Kirby, P., Superbie, C., McLoughlin, P.D., 2020. Science to inform policy: Linking population dynamics to habitat for a threatened species in Canada. J Appl Ecol 57, 1314–1327. <https://doi.org/10.1111/1365-2664.13637>

Kushneriuk, R.S., Rempel, R.S., 2011. LSL - Landscape Scripting Language. Ontario Ministry of Natural Resources, Centre for Northern Forest Ecosystem Research, Thunder Bay, ON.

Lacy, R., Miller, P., Traylor-Holzer, K., 2017. Vortex 10 user’s manual. Conservation Breeding Specialist Group and Chicago Zoological Society, Apple Valley, Minnesota, USA.

McIntire, E.J.B., Chubaty, A.M., Cumming, S.G., Andison, D., Barros, C., Boisvenue, C., Haché, S., Luo, Y., Micheletti, T., Stewart, F.E.C., 2022. PERFICT  A Re‐imagined foundation for predictive ecology.pdf. Ecology Letters Online, 1–7.

MNRF, 2014b. Integrated Range Assessment for Woodland Caribou and their Habitat - The Far North of Ontario 2013 (No. Version 1.1). Ministry of Natural Resources and Forestry - Species at Risk Branch, Thunder Bay, ON.

Novomestky, F., Nadarajah, S., 2016. Package ‘truncdist.’ <http://r.meteo.uni.wroc.pl/web/packages/truncdist/truncdist.pdf>

Rempel, R.S., Carlson, M., Rodgers, A.R., Shuter, J.L., Farrell, C.E., Cairns, D., Stelfox, B., Hunt, L.M., Mackereth, R.W., Jackson, J.M., 2021. Modeling Cumulative Effects of Climate and Development on Moose, Wolf, and Caribou Populations. Jour. Wild. Mgmt. 85, 1355–1376. https://doi.org/10.1002/jwmg.22094
